# Effect of different chitosan molecular weights on microplastic excretion

**DOI:** 10.3389/fnut.2026.1869140

**Published:** 2026-08-31

**Authors:** Di Liu, Soichiro Morikawa, Ryotaro Izumi, Seiji Kurozumi, Masatoshi Kiyose, Muneshige Shimizu

**Affiliations:** 1 Institute of Oceanic Research and Development, Tokai University, Shizuoka, Japan; 2 Graduate School of Science and Technology, Tokai University, Shizuoka, Japan; 3 Department of Fisheries, School of Marine Science and Technology, Shizuoka, Japan; 4 Koyo Chemical Co. Ltd., Umeda Asuka Building, Osaka, Japan

**Keywords:** chitosan, excretion, gut microbiota, intestinal environment, microplastic

## Abstract

**Introduction:**

Microplastic (MP) ingestion poses a considerable health risk to organisms, including humans, and the environment. The previously established average molecular weight of chitosan that promotes MP excretion is approximately 800,000; however, the effects of chitosan with different molecular weights on MP excretion and the intestinal environment remain unclear. This study aimed to clarify the effects of different chitosan molecular weights on MP excretion and gut microbiota.

**Methods:**

Sprague–Dawley rats were fed a diet containing MPs with an average particle size of 50 μm.

**Results:**

Compared with the control group, fecal weight increased in all chitosan groups across different molecular weights (low: 83,000; medium: 673,000; high: 1,877,000). Furthermore, the medium and high molecular weight chitosan groups exhibited a significantly higher MP excretion rate and lower MP retention rate in the gastrointestinal tract. The *16S* rRNA metagenomic analysis revealed a decrease in *Firmicutes* and an increase in *Bacteroidota* and *Proteobacteria* abundance in the high-molecular-weight chitosan group. Additionally, the abundance of beneficial bacterial genera, such as *Akkermansia* and *Bacteroides*, increased. These findings confirm that chitosan with ≥673,000 molecular weight promotes MP excretion. Additionally, chitosan intake may help protect the intestinal environment by improving gut microbiota.

## 1 Introduction

The global plastic production has substantially increased with the widespread use of plastics in industry, agriculture, and daily life (1). Environmental pollution of microplastics (MPs), plastic particles that are less than 5 mm in diameter, is becoming considerably increasing. In 2024, global plastic production reached approximately 430.9 million tons, with widespread applications in food packaging, building insulation, cosmetics, and abrasive cleaners (2). In the environment, plastics break down through various processes, including biodegradation, photodegradation, thermo-oxidative degradation, thermal degradation, and hydrolysis (3).

MPs have been found in rivers, oceans, and the atmosphere (4–6). Owing to their small size, MPs are easily ingested by organisms, including humans, causing concerns regarding the health risks associated with bioaccumulation. Deng et al. (7, 8) reported that continuous oral administration of MPs smaller than 50 μm to mice resulted in their absorption through the gastrointestinal tract and subsequent accumulation in multiple tissues, including the liver and kidneys. Moreover, MPs have been detected in human lungs, feces, blood, and brains (9, 10). These findings raise concerns regarding the potential health impacts on various organisms' MPs that enter the human body via oral ingestion, inhalation, and dermal contact, with primary exposure through food consumption (11–14). Therefore, the rapid excretion of orally ingested MPs from the body is crucial.

Liu and Shimizu (15) investigated the dietary materials that could aid in MP excretion. Rats were co-administered 200 μm polyethylene (PE) MPs along with various indigestible materials that inhibit neutral fat absorption and regulate gut function. For the first time, it was demonstrated that the intake of chitosan derived from crab and shrimp shells promotes MP excretion (15). Furthermore, compared with other indigestible dietary materials, chitosan led to fewer MPs remaining in the gastrointestinal tract and facilitated faster excretion. Consequently, for 200 μm MPs, chitosan may be a useful dietary material for promoting their excretion from the body.

Chitosan is a high-molecular-weight polysaccharide composed of the amino sugar glucosamine. It is a cationic dietary fiber that cannot be degraded by human digestive enzymes. It improves serum cholesterol levels, inhibits fat absorption, and suppresses blood pressure elevation. Furthermore, the molecular weight of chitosan affects its viscosity and influences its bioactivities, such as antioxidant properties and heavy metal adsorption (16–18). Although not explicitly stated in Reference 15, the chitosan used in that study had an average molecular weight of approximately 800,000, which is the established weight known to promote MP excretion; however, the effects of chitosan with different molecular weights remain unclear.

Zhang et al. (19) reported that the ingestion of polystyrene MPs significantly reduces the population of potentially beneficial gut bacteria (19). Additionally, it causes intestinal barrier dysfunction, gut microbiota dysbiosis, and metabolic disorders associated with neuroinflammation (20). Changes in the gut microbiota caused by MP ingestion significantly affect the health of the host organism; therefore, maintaining a favorable intestinal environment is crucial. Although chitosan promotes MP excretion and lower blood cholesterol levels, its relationship with gut flora remains unclear.

Therefore, we aimed to clarify the effects of differences in chitosan molecular weight on MP excretion using three types of chitosan with varying average molecular weights (low, approximately 83,000; medium, approximately 673,000; and high, approximately 1,877,000). Additionally, we aimed to determine the effects of short-term chitosan ingestion on the intestinal environment.

## 2 Materials and methods

### 2.1 Experimental materials

The MP particles used in this study were PE-based. For the 50 μm group, blue-colored particles (product code: BLPMS-1.00) were obtained from Cospheric LLC., with a manufacturer-specified size range of 45–53 μm and an average diameter of 50 μm. The number of particles per gram was estimated to be 1.62 × 10^7^ for this size class.

According to the manufacturer's specifications and Safety Data Sheet (Supplementary SDS-350, rev.3), BLPMS-1.00 particles are solid, spherical, water-insoluble microspheres composed of ≥70% PE (CAS No. 9002-88-4) and ≤ 30% proprietary additives. Their reported density range is 0.99–1.01 g/cm^3^. These density values were used to inform assumptions regarding the physical behavior of the particles within the gastrointestinal matrix.

To verify particle morphology and size, representative samples of the 50 μm MPs were examined using a digital microscope (MRS-T5.1; Alpha Mirage Co. Ltd.) at a total magnification range of 4 × -160 × (optical: 4 × -40 × ; digital zoom: up to 4 × ). The micrographs (Supplementary Figures 1, 2) confirmed that the particles were predominantly spherical and within the expected size distribution. The same microscope system was used for routine particle enumeration in the spiked and recovered fecal samples.

The polymer composition of the 50-μm MPs was confirmed using micro-Fourier transform infrared spectroscopy (Nicolet iN10MX, Thermo Fisher Scientific). Characteristic absorption bands were observed at 2,915, 2,850, 1,465, 730, and 720 cm^−1^, corresponding to the C–H stretching and CH_2_ bending/rocking vibrations typical of PE. These spectra closely match the standard LDPE/HDPE reference patterns, confirming that the particles are primarily composed of PE (Supplementary Figure 3).

Three grades of chitosan—high (FH-80), medium (FM-80), and low (FL-80) molecular weight—were sourced from Koyo Chemical Co., Ltd. (Japan). All chitosan samples were derived entirely from red snow crab (Chionoecetes japonicus Rathbun) harvested in Sakaiminato, Tottori, Japan. According to the manufacturer's analytical data, the physicochemical properties of the respective chitosan grades are as follows:

High molecular weight (FH-80): Weight-average molecular weight (Mw) = 1,877,000 Da; degree of deacetylation (DDA) = 80.5%; viscosity = 130 mPa·s (measured in a solution of 0.5% chitosan and 0.5% acetic acid at 20 °C).

Medium molecular weight (FM-80): Mw = 673,000 Da; DDA = 84.6%; viscosity = 20 mPa·s (measured in a solution of 0.5% chitosan and 0.5% acetic acid at 20 °C).

Low molecular weight (FL-80): Mw = 83,000 Da; DDA = 88.3%; viscosity = 5 mPa·s (measured in a solution of 1.0% chitosan and 1.0% acetic acid at 20 °C).

Regarding solubility, all the above chitosan grades are insoluble in water but can be fully dissolved in dilute acidic solutions, such as acetic acid. According to the manufacturer's supplemental data, the zeta potential of this chitosan was approximately +63 mV at pH 2. Although the zeta potential in the weakly acidic or neutral range was not determined, the surface charge of chitosan is fundamentally governed by the protonation of its amino groups, which directly correlates with the DDA. Therefore, the detailed DDA values provided above serve as a primary indicator of the charge characteristics of the respective chitosan grades in this study.

### 2.2 Experiment 1

#### 2.2.1 Animal experiments

Twenty-four 9-week-old male Sprague–Dawley rats obtained from Japan SLC Inc., were used in this study. The diet composition was based on the AIN-93M formula, the standard diet for rodent experiments according to the American Institute of Nutrition (21). After a 5-day acclimation period on the basal AIN-93M diet, the rats were divided into four groups based on their body weight: control (Group C), low molecular weight chitosan (Group L), medium molecular weight chitosan (Group M), and high molecular weight chitosan (Group H) (*n* = 6 per group). The mice were housed in metabolic cages.

The control diet was prepared by adding 0.0113 g of PE particles with an average diameter of 50 μm (Cospheric LLC; 1.623 × 10^7^ particles/g) to 1 kg of the basal AIN-93M diet (Table 1). For Groups L, M, and H, diets were prepared by replacing a portion of the cornstarch in the control diet with the respective chitosan. These purified diets were continuously administered for 4 days, followed by a 24-h fasting period.

**Table 1:** Feed composition.

Group	C	L	M	H
Cornstarch	465.7	415.7	415.7	415.7
α-Cornstarch	155.0	155.0	155.0	155.0
Low molecular weight chitosan	–	50.0	–	–
Medium molecular weight chitosan	–	–	50.0	–
High molecular weight chitosan	–	–	–	50.0
Milk casein	140.0	140.0	140.0	140.0
Granulated sugar	100.0	100.0	100.0	100.0
Cellulose powder	50.0	50.0	50.0	50.0
Soybean oil	40.0	40.0	40.0	40.0
AIN-93 mineral mix	35.0	35.0	35.0	35.0
AIN-93 vitamin mix	10.0	10.0	10.0	10.0
L-Cystine	1.8	1.8	1.8	1.8
Choline bitartrate	2.5	2.5	2.5	2.5
t-Butylhydroquinone	0.008	0.008	0.008	0.008
MP	–	0.0113	0.0113	0.0113
Total	1,000.0	1,000.0	1,000.0	1,000.0 (g)

The animals were housed in an environment with a room temperature of 23 ± 1 °C, humidity of 35 ± 5%, and 12-h light/dark cycle (light period: 8:00 a.m. to 8:00 p.m.). The diets were provided via pair-feeding and the rats had *ad libitum* access to Milli-Q water. During the test period, the fecal weight, body weight, food intake, and water intake were measured daily. Feces were collected and stored at −20 °C.

On the final day, rats were anesthetized with isoflurane. Organs (heart, liver, kidneys, gastrointestinal tract, and adipose tissue [epididymal and perirenal fat]) were excised and weighed using an electronic balance (SARTORIUS ALA230S). Additionally, whole blood was collected under anesthesia and centrifuged (1,900 × g for 10 min at 4 °C) to obtain plasma. All collected feces, organs, and plasma were stored at −80 °C. This experiment was conducted in accordance with the animal experimentation guidelines after receiving approval from the Animal Experimentation Committee of Tokai University (approval no. 221099).

#### 2.2.2 Measurement of MP count in feces

Cryopreserved feces were thawed at room temperature, homogenized, and 40 mg of the sample was accurately weighed. The feces were added to a 30% H_2_O_2_ solution (FUJIFILM Wako Pure Chemical Corporation) and continuously stirred for 48 h at 60 °C using a hot stirrer (RSH-1DN, AS ONE Corporation). Subsequently, all PE particles were collected onto a polytetrafluoroethylene membrane (Omnipore™, 5 μm × ϕ47 mm) using vacuum filtration (22–25). After the membrane was dried on a clean bench, the PE particles were counted using a digital microscope (LCD Digital Microscope; DIM-03). The number of excreted PE particles per individual was calculated based on the fecal weight. Furthermore, the MP excretion rate in the feces was determined by calculating the number of PE particles in the diet based on food intake.

#### 2.2.3 Measurement of MPs in the gastrointestinal tract

The gastrointestinal tract was cryopreserved after dissection, thawed at room temperature, and separated into distinct sections (stomach, small intestine, and large intestine). These sections were added to a 10% KOH solution (FUJIFILM Wako Pure Chemical Corporation) and stirred for 24 h at 60 °C using a hot stirrer (RSH-1DN, AS ONE Corporation). Subsequently, all PE particles were collected onto a polytetrafluoroethylene membrane (Omnipore™, 5 μm × ϕ47 mm) via vacuum filtration (22–25). After the membrane was dried on a clean bench, PE particles were counted under a microscope. Furthermore, the MP retention rate in the gastrointestinal tract was determined by calculating the number of PE particles in the diet based on food intake.

### 2.3 Experiment 2

#### 2.3.1 Animal experiments

Twenty-four 7-week-old male Sprague–Dawley rats obtained from CLEA Japan Inc., were used in this study. The diet composition was based on the AIN-93M formula, the standard diet for rodent experiments according to the American Institute of Nutrition. After a 5 day acclimation period on the basal AIN-93M diet, the rats were divided into four groups based on their body weight: control (Group C), control + MP (Group CP), high molecular weight chitosan (Group K), and high molecular weight chitosan + MP (Group KP) (*n* = 6 per group). The mice were housed in metabolic cages.

The MP-supplemented diet was prepared by adding 0.0113 g of PE particles with an average diameter of 50 μm (Cospheric LLC; 1.623 × 10^7^ particles/g) to 1 kg of the basal AIN-93M diet. For Group K, the diet was prepared by replacing a portion of the cornstarch in the control diet with high-molecular weight chitosan. For Group KP, the diet was prepared by replacing a portion of the cornstarch in the MP-supplemented diet with high-molecular weight chitosan (Table 2). These purified diets were continuously administered for 8 days, followed by a 24-h fasting period.

**Table 2:** Feed composition.

Group	C	CP	K	KP
Cornstarch	465.7	465.7	415.7	415.7
α-Cornstarch	155.0	155.0	155.0	155.0
High molecular weight chitosan	–	–	50.0	50.0
Milk casein	140.0	140.0	140.0	140.0
Granulated sugar	100.0	100.0	100.0	100.0
Cellulose powder	50.0	50.0	50.0	50.0
Soybean oil	40.0	40.0	40.0	40.0
AIN-93 mineral mix	35.0	35.0	35.0	35.0
AIN-93 vitamin mix	10.0	10.0	10.0	10.0
L-Cystine	1.8	1.8	1.8	1.8
Choline bitartrate	2.5	2.5	2.5	2.5
t-Butylhydroquinone	0.008	0.008	0.008	0.008
MP	–	0.0113	–	0.0113
Total	1,000.0	1,000.0	1,000.0	1,000.0 (g)

The animals were housed in an environment with a room temperature of 23 ± 1 °C, a humidity of 35 ± 5%, and a 12-h light/dark cycle (light period: 8:00 a.m. to 8:00 p.m.). The diets were provided via pair-feeding and rats had *ad libitum* access to Milli-Q water. During the test period, the fecal weight, body weight, food intake, and water intake were measured daily.

On the final day, rats were anesthetized with isoflurane. Organs (heart, liver, kidneys, gastrointestinal tract, and adipose tissue [epididymal and perirenal fat]) were excised and weighed using an electronic balance (SARTORIUS ALA230S). Additionally, whole blood was collected under anesthesia and centrifuged (1,900 × g for 10 min at 4 °C) to obtain plasma. All collected feces, organs, and plasma were stored at −80 °C. This experiment was conducted in accordance with the animal experimentation guidelines after receiving approval from the Animal Experimentation Committee of Tokai University (approval no. 241071).

#### 2.3.2 Measurement of gut microbiota

Feces cryopreserved at −80 °C were transferred to a −25 °C freezer for 12 h. Subsequently, 70 mg of each sample was accurately weighed using an electronic balance (ER-180A, A & D Company, Limited), placed into a bead tube, and mixed with 500 μl RNase-free H_2_O. DNA extraction was performed using the NucleoSpin^®^ DNA Stool kit, MN Bead Tube Holder (Takara Bio Inc.), VORTEX-GENIE^®^ 2 (Scientific Industries), and a refrigerated microcentrifuge.

The V3–V4 region of *16S* rDNA in the extracted nucleic acid samples was amplified using PCR. After attaching the adapter and index sequences, a comprehensive analysis of the gut microbiota was conducted by sequencing the *16S* rDNA using a next-generation sequencer (NextSeq 1,000, Illumina, USA). The obtained data were analyzed using QIIME 2 to determine the relative abundance and α-diversity. Fecal samples were collected on the day before group assignment and 192 h post-assignment.

### 2.4 Statistical analysis

The obtained data were expressed as the Mean ± Standard Error. For comparisons between groups, a one-way analysis of variance (ANOVA) was performed, followed by Bonferroni's multiple comparison test. All statistical analyses were conducted using EZR, a graphical user interface for R. The significance level was set at *p* < 0.05. Comparisons were made among all groups.

## 3 Results

### 3.1 Experiment 1

#### 3.1.1 Changes in body weight and cumulative food intake

**Figure 1:**
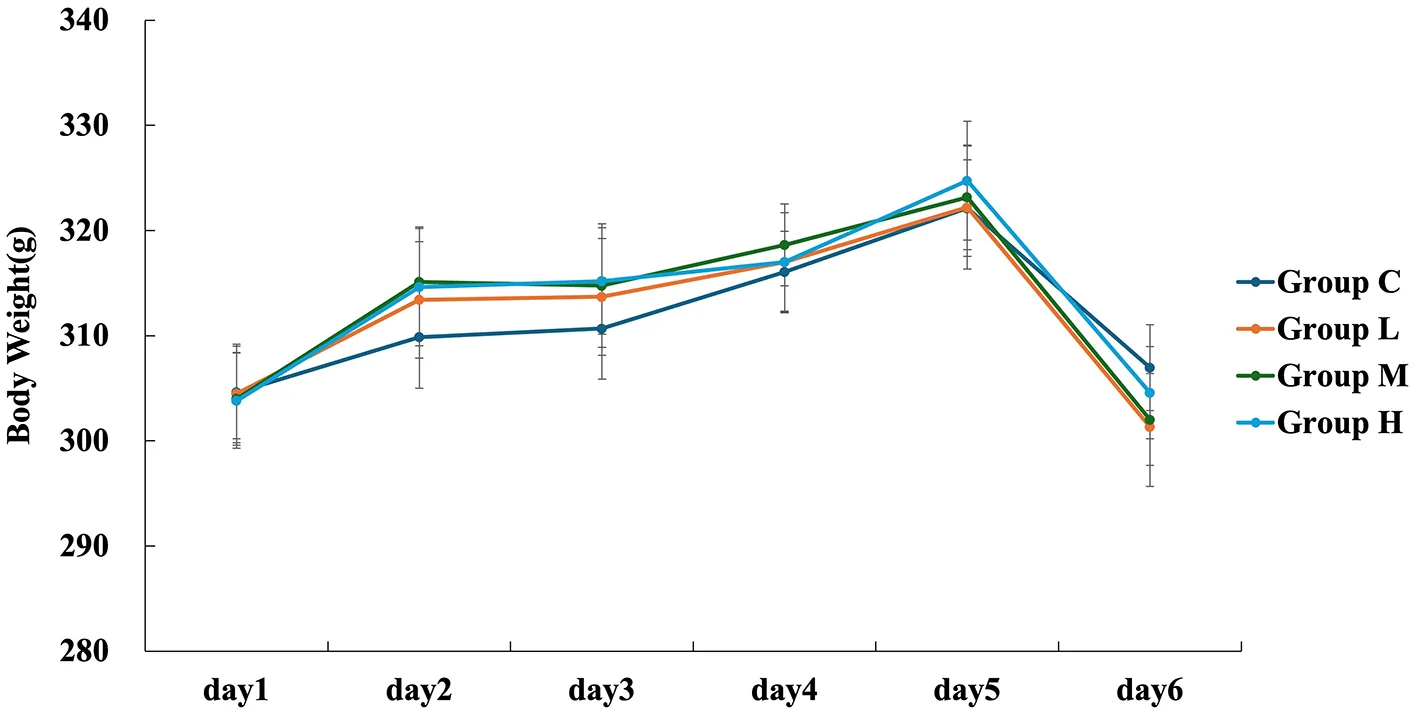
Body weight. Changes in body weight during the test period. No significant differences were observed among the groups. Values are presented as the mean ± SE (*n* = 6 per group).

**Figure 2:**
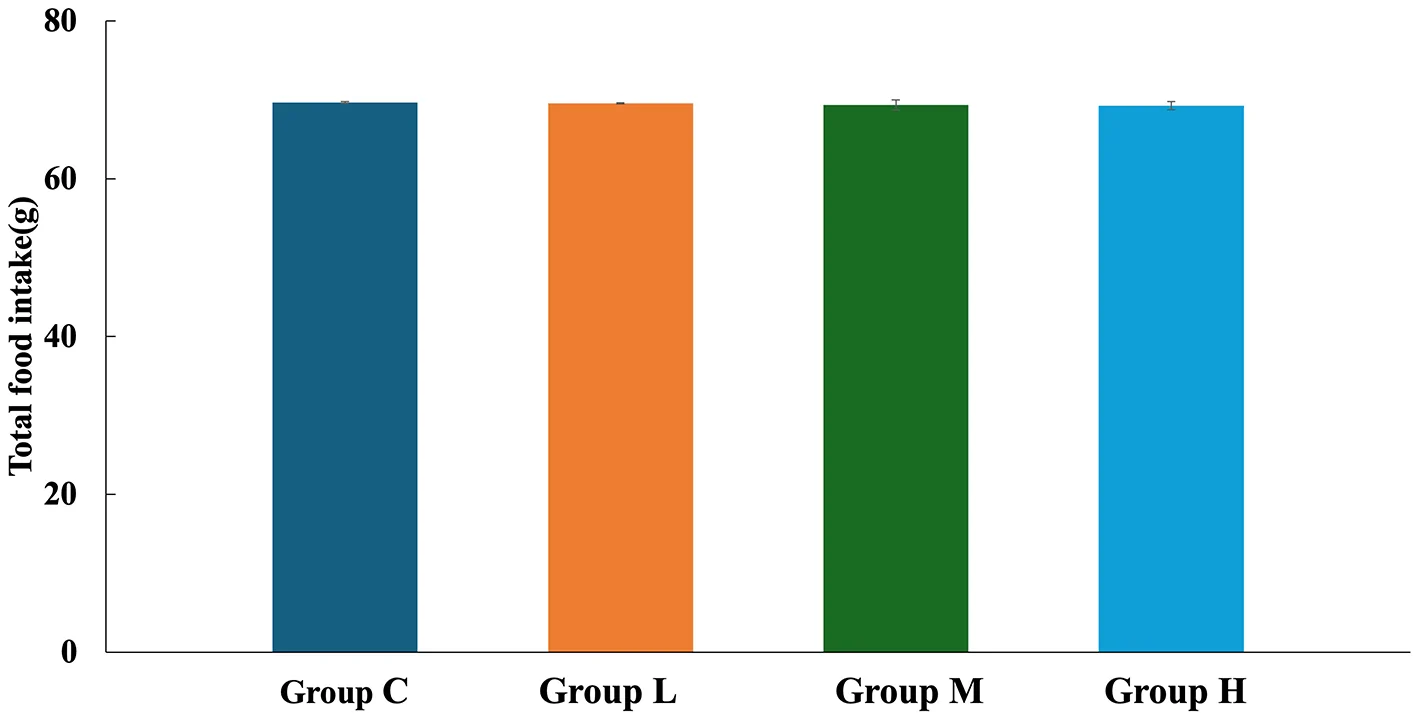
Total food intake. No significant differences were observed among the groups. Values are presented as the mean ± SE (*n* = 6 per group).

Changes in body weight after MP ingestion during the test period are shown in Figure 1 and the total food intake of the MP-supplemented diet over a 4-day period is shown in Figure 2. The average body weight at the start of the experiment was 304.2 ± 2.3 grams. Even after switching to a diet supplemented with MP, body weight continued to increase steadily, and by day 5, the rate of weight gain was similar across all groups. (Group C: 322.1 ± 4.6 g; Group L: 322.2 ± 5.9 g; Group M: 323.2 ± 5.0 g; Group H: 324.7 ± 5.6 g). We fasted for 24 h before the autopsy. In all groups, the average daily food intake remained between 17.3 and 17.4 g, and no animals were excluded because of decreased food intake or other reasons. Additionally, no significant differences in body weight or cumulative food intake were observed between the groups regarding.

#### 3.1.2 Water intake

**Figure 3:**
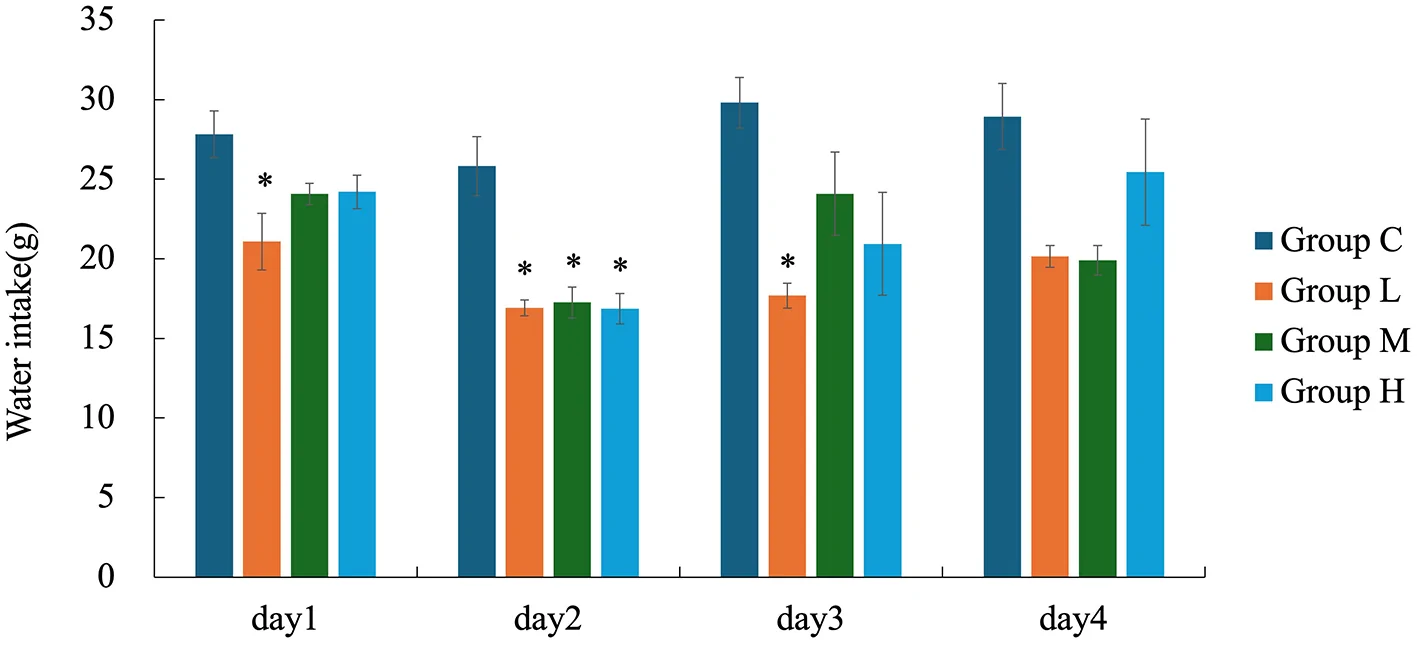
Water intake. Values are presented as the mean ± SE (*n* = 6 per group). *p < 0.05 compared with group C.

Figure 3 presents the daily water intake of the rats over the 4-day test period. Overall, the control group (Group C) maintained the highest water consumption. On Day 2, water intake in all chitosan-fed groups (Groups L, M, and H) was significantly lower than that of Group C. Furthermore, Group L exhibited significantly reduced water intake on Days 1, 2, and 3 compared with the control.

#### 3.1.3 Tissue weights

**Figure 4:**
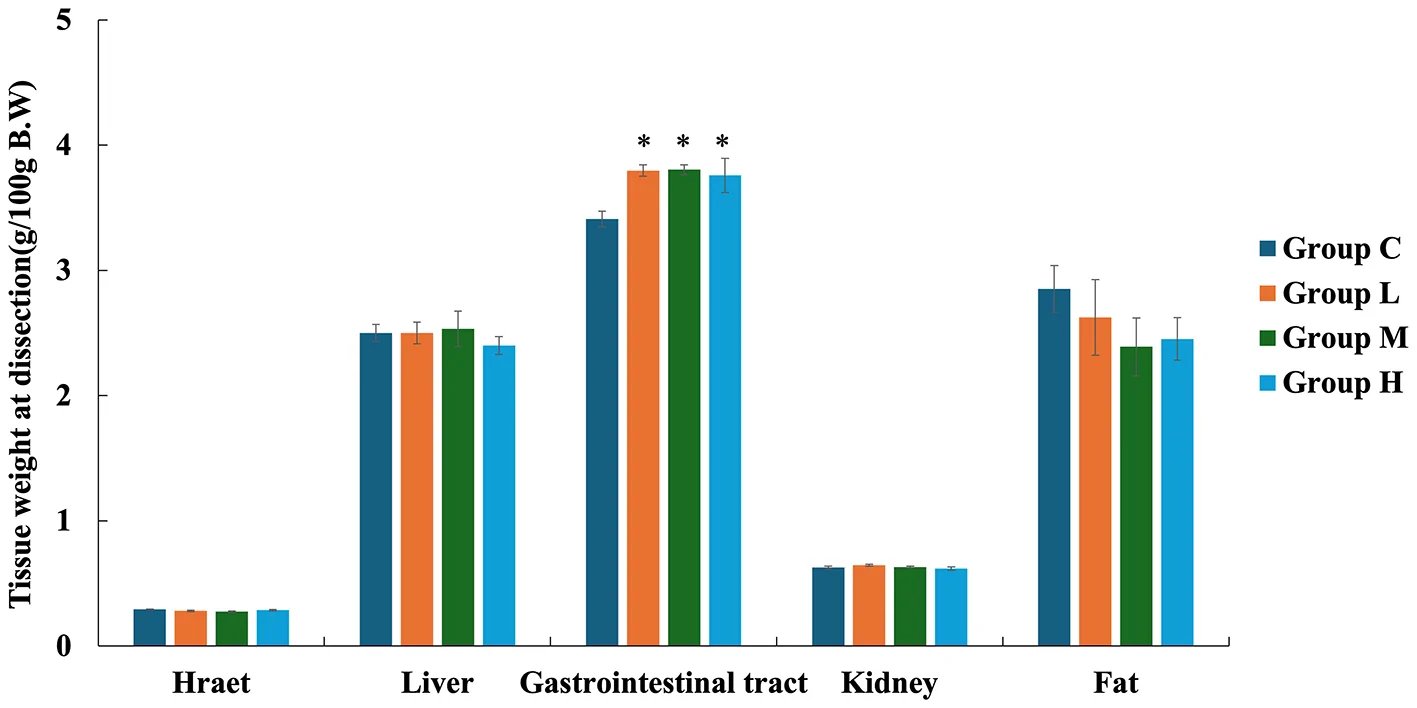
Tissue weight at dissection. Values are presented as the mean ± SE (*n* = 6 per group). “Fat” denotes perirenal fat and epididymal fat. *p < 0.05 represents a significant difference compared with the control group (Group C).

Tissue weights after 4 days of ingesting the MP-supplemented diet are shown in Figure 4. Weights are expressed per 100 g of body weight. For the gastrointestinal tract, Groups L, M, and H showed a significant increase in tissue weight compared with that of Group C. No significant differences in other tissue weights were observed between the groups.

#### 3.1.4 Fecal weight

**Figure 5:**
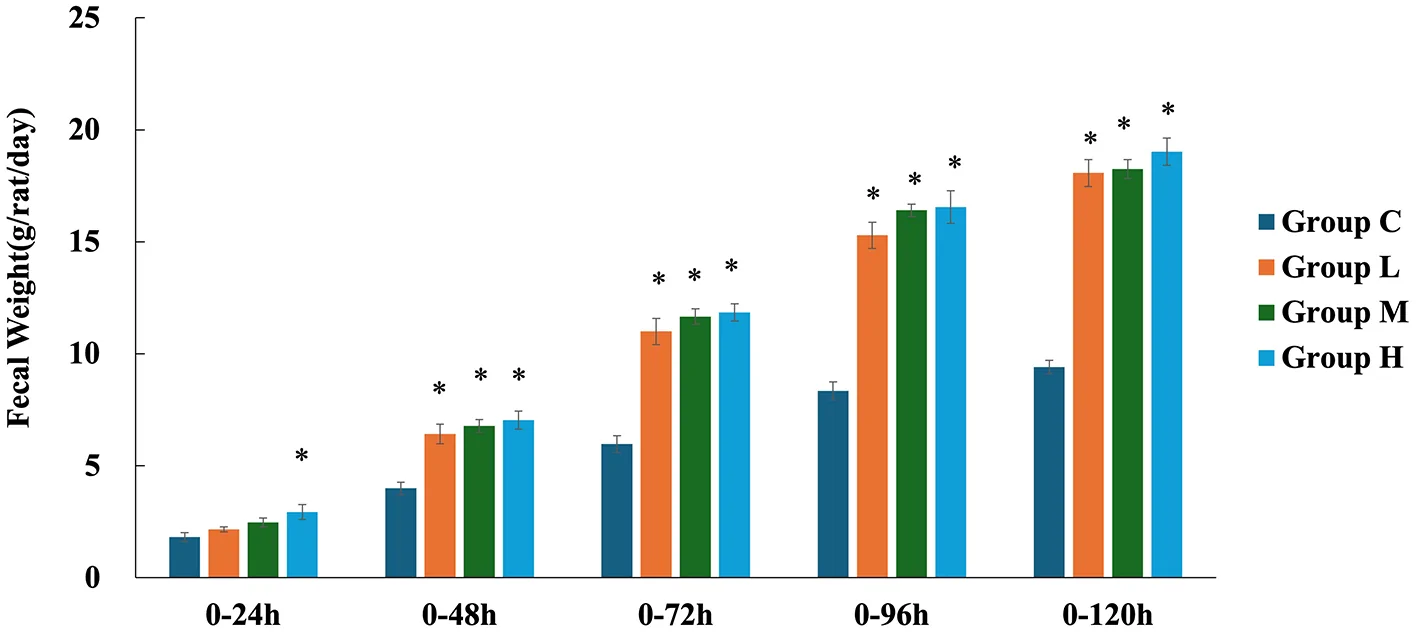
Fecal weight. Diurnal changes were observed from the start of the MP diet until the 120 h (days 1–5). Values are presented as the mean ± SE (*n* = 6 per group). *p < 0.05, represents a significant difference compared with the control group (Group C).

Figure 5 shows the daily changes in cumulative fecal weight from the start of the MP-supplemented diet to day 5. Fecal samples were collected daily in metabolic cages. Differences in fecal weight were observed starting the day after the ingestion of high-molecular weight chitosan. From 0 to 24 h onward, Group H showed a significant increase in fecal weight compared with that of Group C. However, from 0 to 48 h onward, Group L showed a significant increase in fecal weight compared with that of Group M.

#### 3.1.5 MP excretion rate in feces

**Figure 6:**
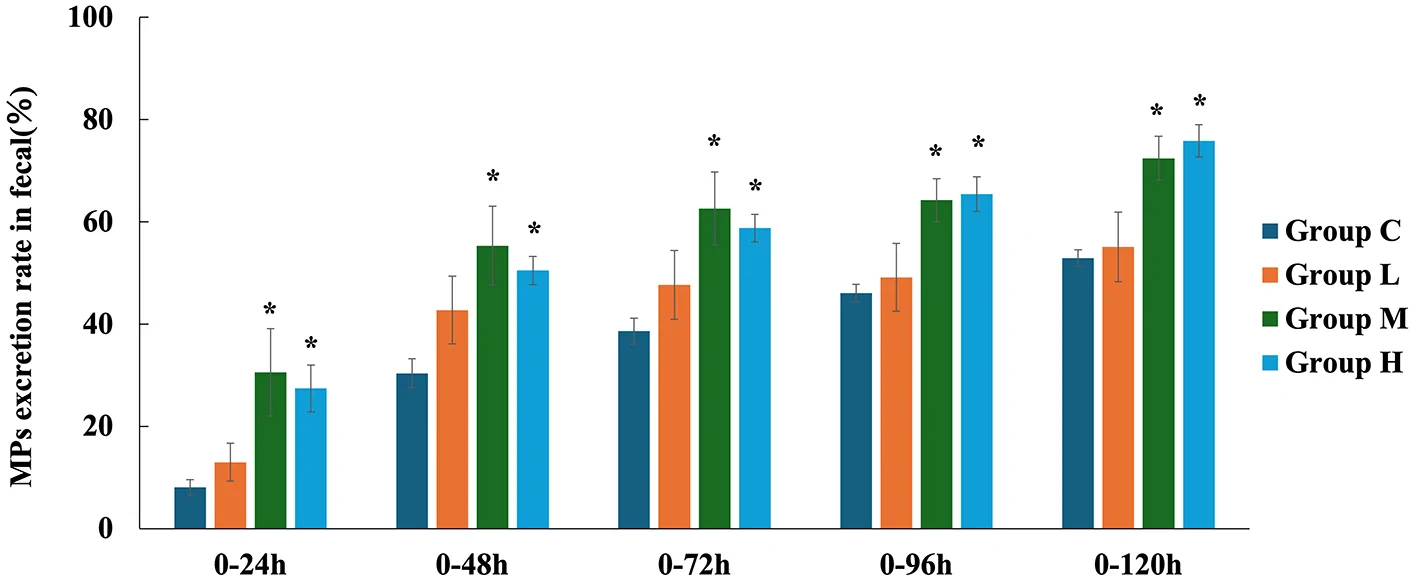
MP excretion rate in fecal matter. The MP excretion rate was calculated as the ratio of the number of MPs ingested from food during the given time period to the number of MPs excreted in feces. Values are presented as the mean ± SE (*n* = 6 per group). *p < 0.05, represents a significant difference compared with the control group (Group C).

The MP excretion rate in the feces from the start of the MP-supplemented diet until day 5 is shown in Figure 6. The MP excretion rate in Group C was 8.1 ± 3.7% at 0–24 h and 52.9 ± 3.9% at 0–120 h after MP ingestion. At all-time points from 0 to 24 h onward, Groups M and H exhibited significantly higher MP excretion rates than those of Group C (at 0–120 h: group M, 72.4 ± 10.5%; group H, 75.8 ± 7.8%).

**Figure 7:**
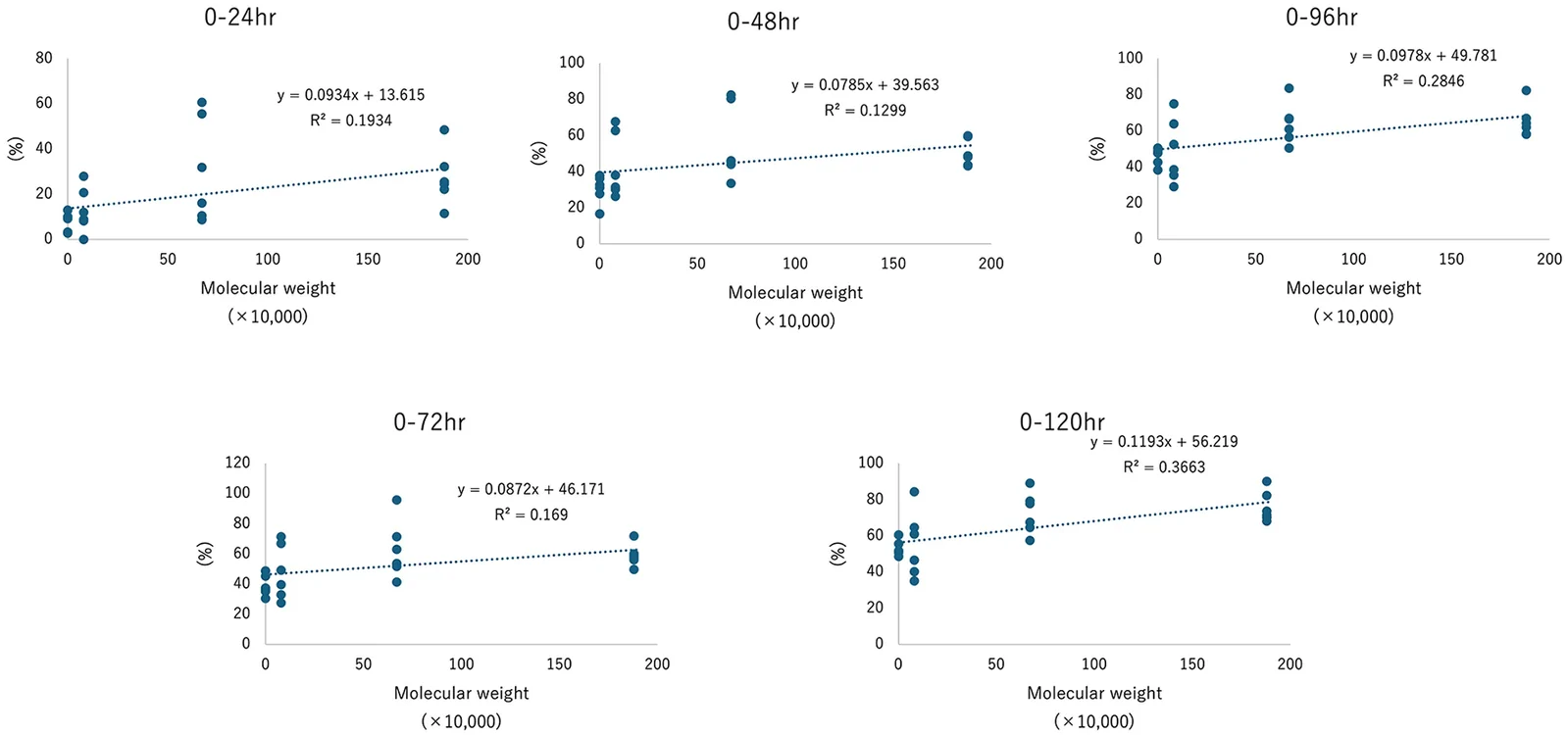
Relationship between molecular weight of chitosan and MP excretion rate (*n* = 24).

Figure 7 shows the relationship between the molecular weight of chitosan and MP excretion rate across each observation period from 0 to 24 h and 0 to 120 h. Given the sample size (*n* = 22), these relationships do not satisfy statistical significance criteria and should be interpreted as exploratory trends with minimal explanatory value. A weak positive trend was observed between the two variables at 0–24 h (R^2^ = 0.1934) and the R^2^ value gradually increased as observation time increased. The highest coefficient of determination was observed at 0–120 h (R^2^ = 0.3663), indicating a potential tendency for the relationship between the chitosan molecular weight and MP excretion rate to become clearer over time. Furthermore, while the regression line slope was positive across all observation periods, this suggests an exploratory pattern rather than definitively confirming that the MP excretion rate increased with increasing chitosan molecular weight.

#### 3.1.6 MP retention rate in the gastrointestinal tract

**Figure 8:**
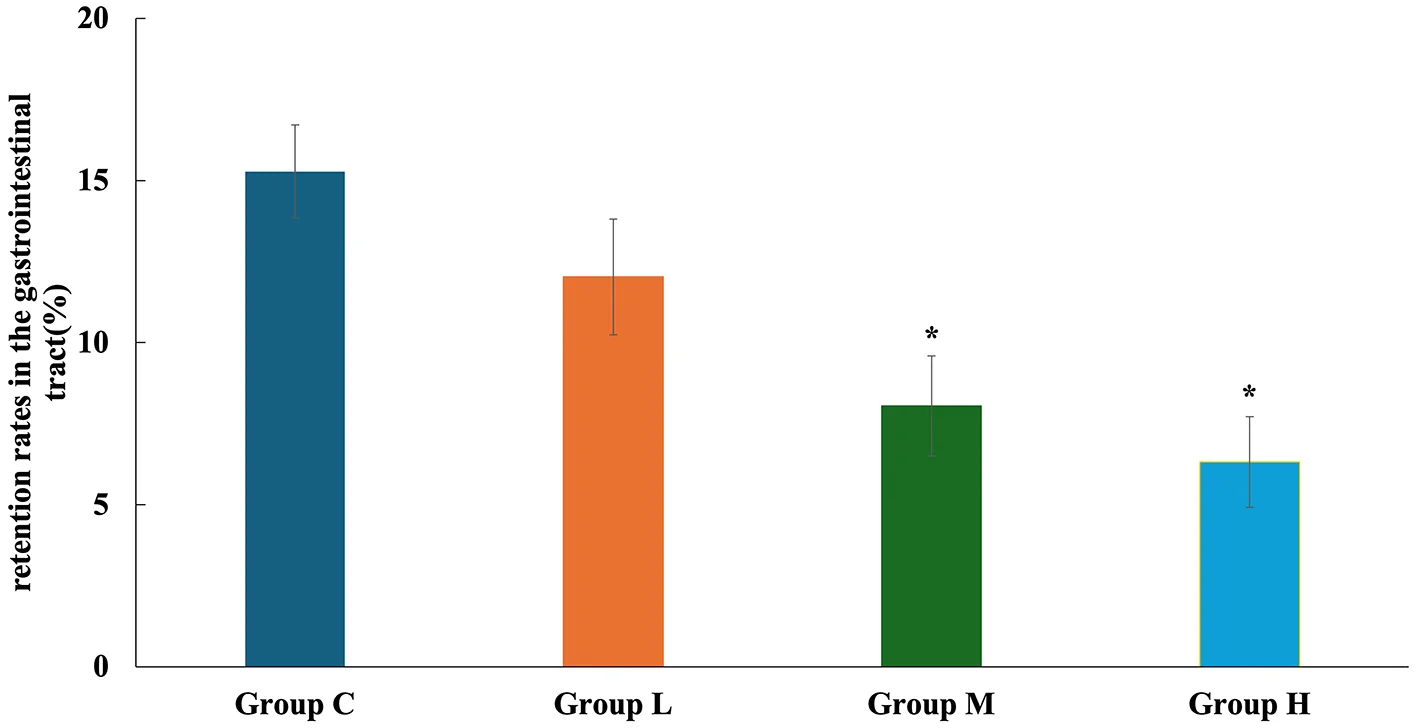
MP excretion rate in the gastrointestinal tract. Values are presented as the mean ± SE (*n* = 6 per group). *p < 0.05 represents a significant difference compared with the control group (Group C).

MP retention rates in the gastrointestinal tract are shown in Figure 8. The rates were 15.3 ± 1.4% for Group C, 12.0 ± 1.8% for Group L, 8.0 ± 1.5% for Group M, and 6.3 ± 1.4% for Group H. Groups M and H showed significantly lower values compared with those of Group C. Furthermore, Group H showed significantly lower values compared with those of Group L.

### 3.2 Experiment 2

#### 3.2.1 Changes in body weight and cumulative food intake

**Figure 9:**
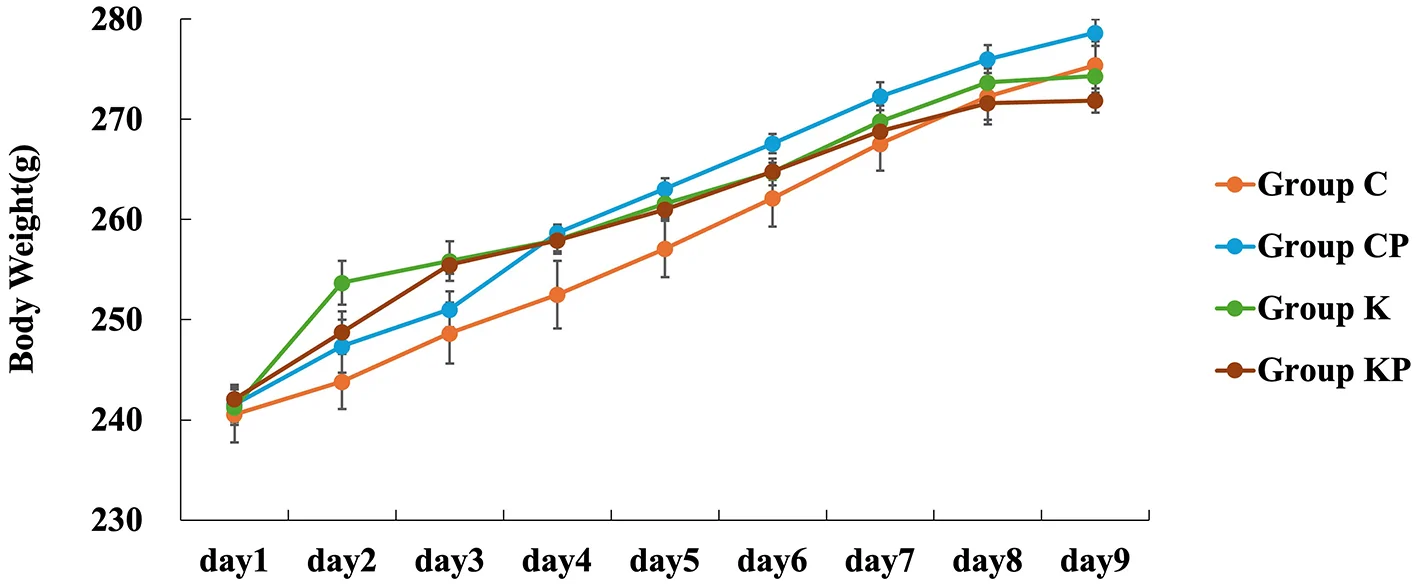
Body weight. Changes in body weight during the test period. No significant differences were observed among the groups. Values are presented as the mean ± SE (*n* = 6 per group).

Changes in body weight after MP ingestion during the test period are shown in Figure 9. The average body weight at the time of grouping was 241.4 ± 1.0 g. Body weight continued to increase steadily even after switching to the MP-supplemented diet, showing a similar increase across all groups after 8 days (Group C: 275.4 ± 2.4 g; Group L: 278.7 ± 1.3 g; Group M: 274.3 ± 1.7 g; Group H: 271.9 ± 1.2 g). In all groups, the average daily food intake was 17.4 g, and no animals were excluded for decreased food intake or other reasons. No significant differences in body weight or cumulative food intake were observed between the groups.

#### 3.2.2 Tissue weights

**Figure 10:**
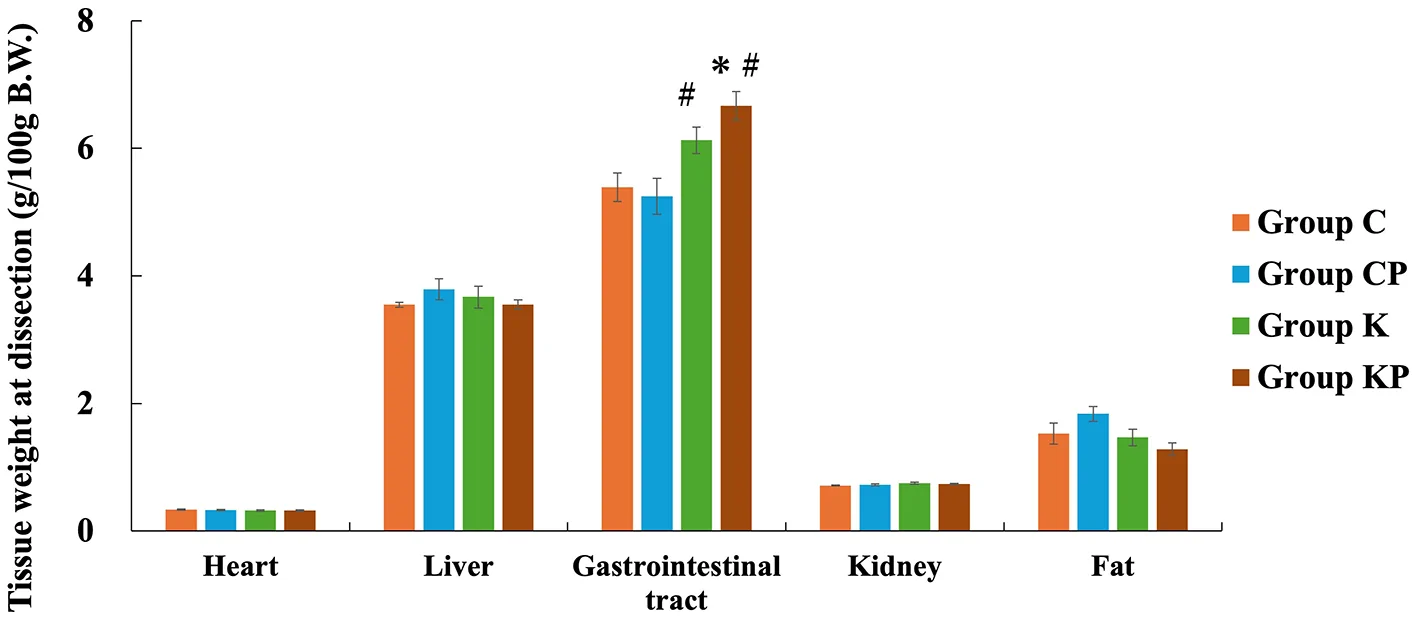
Tissue weight at dissection. Values are presented as the mean mean ± SE (*n* = 6 per group). “Fat” denotes perirenal fat and epdidymal fat. *p < 0.05 represents a significant difference compared with the control group (Group C). ^#^*p* < 0.05 represents a significant difference compared with the control + MP group (Group CP).

The tissue weights per 100 g of body weight at the time of dissection are shown in Figure 10. For the gastrointestinal tract, Group KP showed a significantly higher tissue weight than that of Group C. No significant differences in other tissue weights were observed between the groups.

#### 3.2.3 Fecal weight

**Figure 11:**
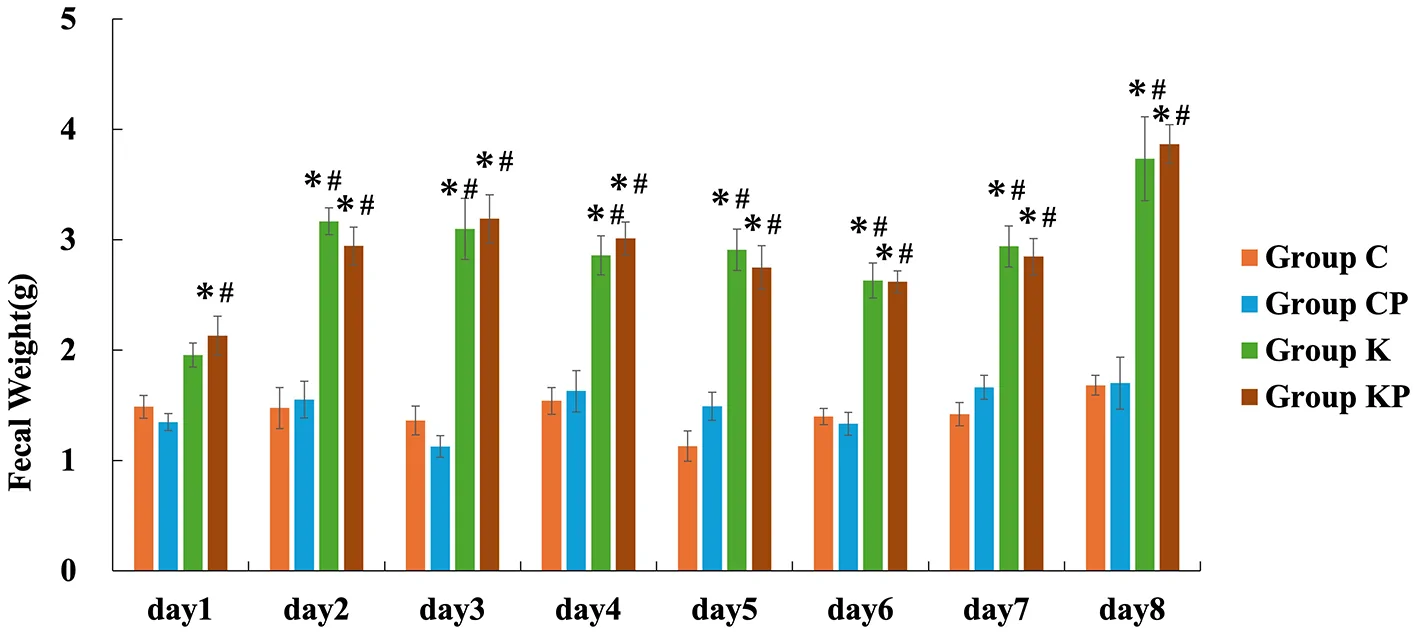
Fecal weight. Diurnal changes were observed from the start of the MP diet until days 8. Values are presented as the mean mean ± SE (*n* = 6 per group). *p < 0.05, represents a significant difference compared with the control group (Group C). ^#^*p* < 0.05 represents a significant difference compared with the control + MPgroup (Group CP).

Figure 11 shows the changes in fecal weight from the start of the MP-supplemented diet to day 8. From 0 to 24 h onward, Groups K and KP showed a significant increase in fecal weight compared to that of Group C. Furthermore, Groups K and KP exhibited a significant increase in fecal weight compared to that of Group CP.

#### 3.2.4 Effects on gut microbiota

##### 3.2.4.1 Relative abundance and major bacterial species

**Figure 12:**
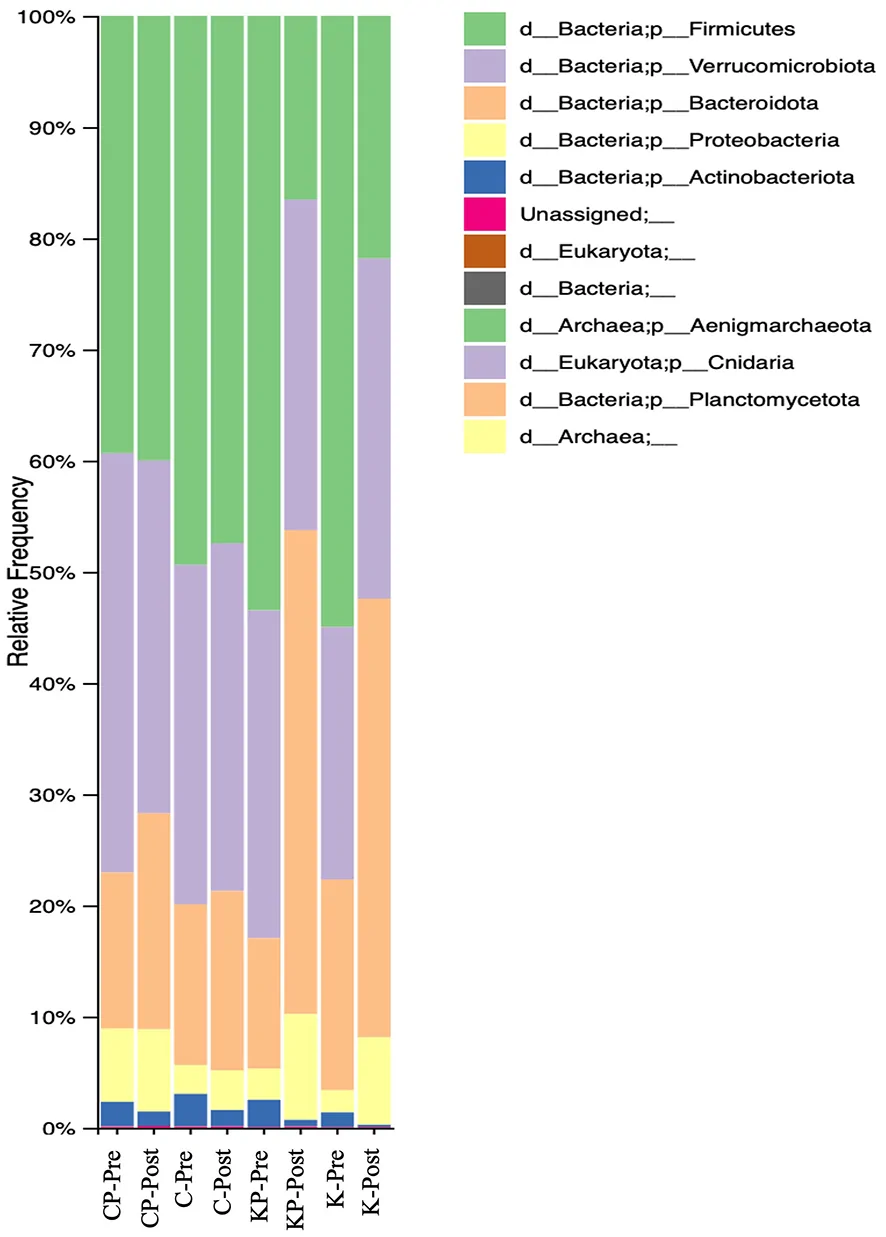
Relative abundance of the gut microbiota at the phylum level. Stacked bar charts illustrate the relative frequency of the major bacterial phyla in each group (Groups C, CP, K, and KP) before (pre) and after (post) the intervention. Each color represents specific bacterial phylum, with the major constituent phyla being *Firmicutes, Verrucomicrobiota, Bacteroidota, Proterobacteria*, and *Actinobacteriota*.

Figure 12 shows the relative abundance of the gut microbiota at the phylum level. The major phyla were *Firmicutes, Verrucomicrobiota, Bacteroidota, Proteobacteria*, and *Actinobacteriota*. In Group C, no significant changes in microbiota composition were observed throughout the experimental period. In Group CP, which ingested MPs, there was a tendency for the relative abundance of *Verrucomicrobiota* to decrease and *Bacteroidota* to increase. Conversely, in Groups K and KP, which ingested chitosan, *Firmicutes* and *Actinobacteriota* markedly decreased after ingestion, whereas *Bacteroidota* and *Proteobacteria* increased in both groups.

Table 3 shows the relative abundances of the major bacteria at the genus level. The major genera were *Akkermansia, Bacteroides, Alloprevotella*, and *Romboutsia*. In Group C, no major changes in microbiota composition were observed throughout the experimental period. In Group CP, *Akkermansia* tended to decrease, whereas *Bacteroides* and *Alloprevotella* increased after MP ingestion. However, the changes were more pronounced in Groups K and KP. In both groups, *Bacteroides* and *Alloprevotella* substantially increased, whereas *Romboutsia* markedly decreased.

**Table 3:** Major genera (%).

Group	C-pre	C-post	CP-pre	CP-post	K-pre	K-post	KP-pre	KP-post
Akkermansia	26.6	27.4	34.2	24.9^*^	19.3	26.9	27.2	26.8
Bacteroides	5.4	6.7	6.5	9.2	6.8	17.5^*^	6.1	16.1^*^
Alloprevotella	2.8	4.4	3.1	5.4	6.4	12.2	5.2	14.7^*^
Romboutsia	8.8	2.8	6.6	4.7	12.3	0.2^*^	10.5	0.2^*^

Data are presented as the relative abundance (%) of selected gut microbiota genera. C, control group; CP, microplastics group; K, chitosan group; KP, chitosan + microplastics group. Pre indicates pre-intervention (baseline), and Post indicates post-intervention. ^*^p < 0.05 represents a significant difference compared with the Pre group in each group.

##### 3.2.4.2 α-Diversity

**Figure 13:**
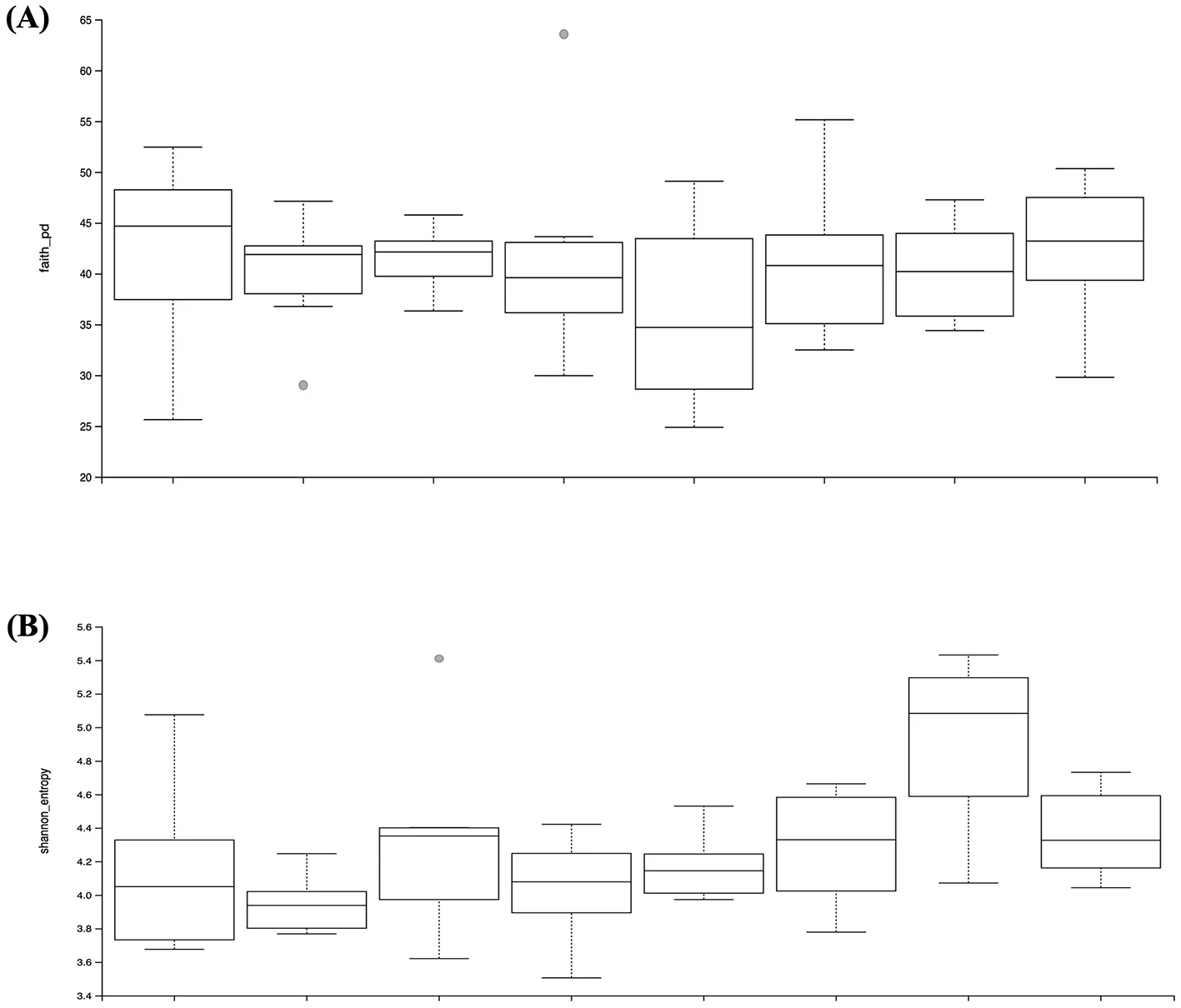
**(A)** Alpha diversity of the gut microbiota evaluated by Faith's phylogenetic diversity. **(B)** Alpha diversity of the gut microbiota evaluated by the Shannon index. Box plots show Faith's PD and Shannon index values for each group (Groups C, CP, K, and KP) before (pre) and after (post) the intervention (*n* = 6 per group). The horizontal line within each box represents the median, and the lower and upper boundaries of the boxes indicate the first and third quartiles, respectively.

The results before the start of the test diet ingestion and on day8 post-ingestion are shown in Figures 13A, B. Regarding Faith's PD, an increasing trend was observed in Group KP, from 36.0 before grouping to 41.2 at day8. However, no major changes were observed in the other groups (Groups C, CP, and K) throughout the study period. For the Shannon index, although a slight decrease (from 4.9 to 4.4) was observed in Group K, no significant differences were observed between the groups or between the pre- and post-ingestion time points. These results suggest that under the present experimental conditions, there is no significant impact on the α-diversity of the gut microbiota.

## 4 Discussion

In this study, we investigated the effects of ingesting chitosans with varying molecular weights on MP excretion by administering 50 μm PE particles to rats via a purified diet for 5 days. The results demonstrated that medium-molecular-weight chitosan (approximately 673,000) promoted MP excretion, whereas low-molecular-weight chitosan (approximately 83,000) did not. Liu and Shimizu (15) reported that the fecal MP excretion rate was significantly higher in a group fed chitosan with an average molecular weight of 800,000 than in a control group. In the present study, Group M, which received chitosan with a similar molecular weight, exhibited a comparable MP excretion rate. Furthermore, the analysis of MPs remaining in the gastrointestinal tract revealed significantly lower values in Groups H and M than in Group C, which is consistent with the excretion data.

In a previous study, fecal samples were digested using 10% KOH, and an MP spike-and-recovery test yielded a recovery rate of 81.5% (26). In the present study, the recovery rates from the MP spike-and-recovery tests were 89.2% for feces and 82.3% for the gastrointestinal tract. When recalculating the fecal MP excretion rates based on these recovery rates, the adjusted values were 59.3% in Group C, 61.8% in Group L, 80.8% in Group M, and 86.0% in Group H. Similarly, the retention rates in the gastrointestinal tract were 18.6% in Group C, 14.6% in Group L, 9.8% in Group M, and 7.7% in Group H. The sum of the fecal MP excretion rate and the gastrointestinal retention rate was 77.9% for Group C, 76.4% for Group L, 90.6% for Group M, and 93.7% for Group H. Given that the accumulation of 50 μm MPs in internal organs such as the liver or kidneys is rarely reported in the literature, and over 90% of the MPs in Groups M and H were detected in the feces, and gastrointestinal tract combined, the movement of these MPs appears to be primarily confined within the gastrointestinal lumen. This limited translocation aligns with recent findings showing that larger plastic particles are effectively blocked by the intestinal epithelial barrier (27, 28). Therefore, the physical trapping effect of chitosan operates within a space where most ingested MPs are naturally restricted.

Chitosan ingestion increased fecal weight even in the presence of MPs. A comparison among Groups L, M, and H revealed no significant differences, indicating that variations in the molecular weight of chitosan do not notably alter overall fecal mass. However, while total fecal weight remained similar among these three groups, their respective MP excretion profiles displayed molecular-weight-dependent variations. This divergence indicates that the effects of different molecular weights are not solely mediated by a simple increase in fecal bulk. Instead, variations in molecular weight influence intraluminal dynamics, such as mucosal adhesion or retention times, thereby altering MP behavior through mechanisms beyond generic mechanical expansion. Although an increase in fecal bulk helps eliminate MPs mechanically, the effects observed in this study involve a specificity unique to chitosan. In our previous trials, other dietary materials such as indigestible dextrin and lactosucrose did not promote MP excretion (15), demonstrating that simple fecal expansion or prebiotic action alone is insufficient for MP clearance. However, apart from these substances, we have not evaluated other common bulk-forming fecal expanders. This represents a limitation of our study, and further comparative research is needed to fully clarify this potential specificity.

In this experiment, an exploratory trend between the chitosan molecular weight and MP excretion rate appeared to become more pronounced over time. These results suggest that differences in the molecular weight of chitosan might potentially influence MP dynamics within the gastrointestinal tract, such as hypothesized changes in retention time and mucosal adhesion. These factors could contribute to variations in the cumulative excretion amount over time. However, it must be explicitly noted that direct measurements of intestinal transit (such as the charcoal test, bead transit, or stomach emptying) and *in vivo* luminal viscosity were not evaluated in this study. Therefore, we cannot make definitive causal assertions, and these interpretations regarding transit times and viscosity should be viewed as hypotheses that require further investigation.

The large increase in fecal weight and gut weight in the chitosan groups is important to consider alongside the water intake data. Because chitosan absorbs water and forms gels (27), one might expect that these weight increases were due to drinking more water. However, our data show that water intake in the chitosan-fed groups was generally lower than or similar to the control group during the study (Figure 3). Therefore, the increased gut and fecal weight do not seem to be caused by drinking extra water. Instead, these findings suggest that chitosan has a strong ability to hold onto the moisture already inside the gut. This property is consistent with previous studies showing that chitosan interacts with gut contents to alter their physical characteristics (28), thereby expanding the gut contents and increasing fecal weight without increasing water consumption.

Furthermore, this weight buildup in the gut explains our overall body weight results. A previous study in which rats were fed 5 μm and 20 μm PE particles for 28 days (0.5 mg/day) reported a significant decrease in relative liver weight (7). In contrast, the present study involved a short-term MP ingestion period of 5 days, which accounts for the absence of significant effects on tissue weight. Additionally, the higher gastrointestinal tract weight observed in the chitosan groups compared to Group C is attributed to the greater mass of gastrointestinal contents. Furthermore, despite the high dose of chitosan administered in this study (2.7 g/kg/day), no decrease in body weight was observed. The difference between our results and the study by Bondiolotti et al. (29) is likely due to several factors. First, our 5-day study duration may be insufficient to induce persistent systemic weight loss. Second, both food intake and diet composition differed between the studies; the daily food intake of our rats was higher than that reported by Bondiolotti et al. (1.6 g/kg/day vs. their reported value), and the specific nutritional composition of the feed was also different. These dietary variations may have provided sufficient energy to counteract the potential weight-reducing effects of chitosan. Lastly, an acute physiological adaptation, characterized by the increased mass of gastrointestinal contents in the chitosan groups, temporarily counterbalanced any subtle reductions in body mass. The heavier, water-rich gut contents likely offset minor weight losses in other body tissues, explaining why total body weight did not change significantly even with high doses of chitosan. While recent *in vitro* research indicates that chitosan's molecular weight and viscosity are key factors for trapping MPs (27), *in vivo* comparative studies remain limited. Therefore, future research should incorporate comprehensive information on active ingredients, including their purity and molecular weight.

Another important question is whether chitosan functions exclusively for PE microplastics or if it is also effective for other polymers, such as polystyrene (PS). While this study focused on PE, the mechanism of action—trapping particles within a gel network—suggests that chitosan may interact with other types of plastics. Casella et al. (27) evaluated how different environmental plastics behave, including PS particles. Because chitosan forms a gel based on the general physiological conditions inside the gut rather than a specific chemical reaction with PE, it is possible that it could also facilitate the clearance of other common plastics like PS. However, different plastics possess distinct sizes and surface properties. Therefore, further studies are required to verify the efficacy of chitosan across a wider range of plastic types.

It is also necessary to consider whether chitosan exerts similar effects on nanoplastics (NPs). Compared to microplastics, NPs are much smaller, allowing them to cross the gut barrier more readily and potentially induce greater biological effects. Because chitosan primarily remains inside the intestinal lumen and is not absorbed systemically, it may help trap NPs while they are still within the digestive tract, thereby limiting the amount of NPs that enter the bloodstream. However, once NPs translocate through the gut wall into other tissues, chitosan is unlikely to affect them. Therefore, the protective effects of chitosan against NPs may be more limited compared to microplastics, and further research is needed to clarify how chitosan interacts with these smaller particles.

Beyond altering these physical excretion dynamics, the physical retention of chitosan gels and trapped MPs within the digestive tract is highly likely to modulate the biological environment of the gut. Subsequently, we investigated the effects of MP and chitosan ingestion on the gut microbiota through *16S* rRNA metagenomic analysis of feces. The control group showed a stable microbiota composition dominated by *Firmicutes*, whereas the chitosan-ingested groups exhibited a decrease in *Firmicutes* and an increase in *Bacteroidota* and *Proteobacteria*. This *in vivo* study provides initial evidence that the ingestion of high-molecular weight chitosan increases fecal weight, promotes MP excretion, and influences the short-term balance of short-chain fatty acid-producing bacteria in the gastrointestinal tract.

At the genus level, an increase in Bacteroides and Alloprevotella, and a decrease in Romboutsia were consistently observed following the ingestion of high-molecular-weight chitosan. An increasing trend in Akkermansia abundance was also observed in some groups. Akkermansia is associated with intestinal mucosal barrier function and metabolic regulation, and its abundance typically increases with the intake of indigestible polysaccharides and dietary fiber (30–34). Therefore, this study indicates that chitosan, similar to other dietary fibers, modulates the gut microbiota profile.

Furthermore, the changes observed at the phylum level, specifically the decrease in Firmicutes and the increase in Bacteroidota, are consistent with findings from previous reports indicating that the Firmicutes/Bacteroidota ratio is often elevated in individuals with obesity and lower in those with normal weight (35). This suggests that the ingestion of high-molecular-weight chitosan shifts the composition of the gut microbiota toward a profile associated with altered fat metabolism. Even during MP ingestion, chitosan intake altered the gut microbiota composition by increasing the relative abundance of bacterial genera such as Akkermansia and Bacteroides. When MP ingestion disrupts the balance of the gut flora, the ability of chitosan to maintain these bacterial groups represents a noteworthy physiological response. However, future studies should increase the sample size and incorporate metabolite-level analyses to further investigate the relationship between changes in the microbiota and host physiological functions.

Regarding alpha diversity, Faith's PD showed a slightly increasing trend in the gut microbiota of Group KP, but no major changes were observed in the other groups. The Shannon index showed a slight decreasing trend in Group K; however, overall, no significant differences were found between the groups. This slight decreasing trend in the Shannon index may reflect a minor loss of evenness within the microbiota, potentially due to the dominance of certain bacterial species (such as the increase in the Bacteroidota phylum and Bacteroides genus). These results suggest that under the conditions of this experiment, characterized by short-term and low-dose MP ingestion, the impact on the overall diversity of the gut microbiota was limited.

While these findings suggest that chitosan selectively affects specific components of the microbiota without significantly disrupting the overall ecological balance, it is important to acknowledge that 16S rRNA sequencing only provides compositional data. Therefore, our interpretations regarding the modification of the intestinal environment and the activation of metabolism-related bacteria remain observational. Future studies incorporating metatranscriptomics, metabolomics, and direct functional assays are required to validate these proposed mechanisms.

We acknowledge that the doses of both MPs and chitosan used in this study are higher than typical daily exposures in real-life human scenarios. Human exposure to environmental MPs is usually long-term and occurs at much lower concentrations. However, we implemented this high-dose, short-term plan (a 5-day exposure period) as an accelerated model to detect clear physiological responses and clearance trends within a limited timeframe. Importantly, although our setup serves as an accelerated model, the total amount of MPs administered and ingested in this study remains lower than the levels frequently utilized in many other MP investigations. This indicates that our experimental design is relatively conservative compared to existing literature. Therefore, while these doses represent an upper-bound experimental scenario rather than daily dietary habits, they provide valuable insights into the protective efficacy of chitosan. Future long-term studies utilizing lower, more realistic doses are necessary to confirm these findings.

Importantly, the short timeframe of this experiment represents a limitation. This brief duration restricts our ability to evaluate persistent physiological changes, stable microbial alterations, or long-term metabolic adjustments. While our results highlight critical acute responses, future research must establish long-term ingestion models. These subsequent studies should incorporate larger sample sizes, varying doses of MPs, different chitosan molecular weights, and metabolite-level analyses to fully clarify the lasting impacts on the gut microbiota and host physiological functions.

In conclusion, chitosan promotes the excretion of 50 μm PE microplastics in a molecular-weight-dependent manner. Additionally, it modulates the gut microbiota composition, specifically by altering the relative abundance of bacterial genera such as Akkermansia and Bacteroides. Future studies focusing on long-term ingestion trials and metabolite-level analyses are required to elucidate the underlying mechanisms of these functional effects of chitosan in greater detail.

## Data Availability

The original contributions presented in the study are publicly available. This data can be found here: https://www.ncbi.nlm.nih.gov/sra/PRJNA149718.
